# Internal Neurolysis of the Common Peroneal Nerve With Lower Extremity Flexion Increases Likelihood for Direct End-End Nerve Repairs

**DOI:** 10.7759/cureus.10355

**Published:** 2020-09-10

**Authors:** Michelle R Jennette, Russell Payne, Elias Rizk

**Affiliations:** 1 Cellular and Molecular Physiology, Penn State Health Milton S. Hershey Medical Center, Hershey, USA; 2 Neurosurgery, Penn State Health Milton S. Hershey Medical Center, Hershey, USA

**Keywords:** common peroneal nerve, knee flexion, end-end repair, compression, foot drop

## Abstract

Introduction

An understanding of common peroneal neuropathy is essential to improving current surgical techniques and overcoming limitations of permanent common peroneal nerve (CPN) damage, which negatively impacts quality of life. This study focuses on quantifying additional nerve length after CPN internal neurolysis with varying degrees of lower extremity flexion, in order to improve end-end nerve repairs in the setting of injury with a significant gap.

Materials and methods

Full and partial neurolysis dissections were performed on salt- and formaldehyde-perfused cadavers. For accurate measurement of additional length obtained, all dissections were palpated at the fibular head and CPNs transected proximally at the distal fascial border. We chose this site to measure the nerve length obtained because it is the common site for CPN injury and the most clinically applicable. The amount of nerve length gained from neurolysis alone was measured at the location of the common peroneal nerve passing deep to peroneus longus before and after being transected. Nerve length after neurolysis was measured in cm at 10° increments, using a protractor starting at 90° and ending at 0°.

Results

Both formaldehyde- and salt-perfused cadavers have a significant difference in nerve length obtained after neurolysis at each angle of measurement. This is seen for both dissection groups with p-values <0.05.

Conclusions

In this study, we found a direct statistically significant relationship between extent of neurolysis and increased angle of measurement, to amount of addition nerve length obtained in both neurolysis groups despite fixation type.

## Introduction

Common peroneal nerve (CPN) injury is the most common mononeuropathy of the lower extremity, resulting in weakness of the ankle flexors and evertors [1-3]. CPN injury frequently occurs at the knee, just proximal to the fibular head by iatrogenic causes or secondary to trauma [1,4,5]. Although CPN injuries may resolve spontaneously and without intervention, long-term compression or trauma can result in irreversible damage such as impaired gait, loss of ankle eversion, dorsal foot sensory loss, pain, paresthesia, and foot drop [1,5]. Currently, permanent damage caused by CPN injury is treated with physical therapy, ankle-foot orthosis (AFO), or operative intervention [2,3,6]. Operative techniques include nerve interventions such as neurolysis, end to end repair and interpositional nerve grafting. Nerve interventions include open procedures such as internal neurolysis, which liberates the nerve from surrounding tissue, allowing additional length or decompression to occur [7,8]. However, if damage to the nerve is severe, interventions may include more invasive techniques such as interpositional nerve grafts or end-end repairs. Interpositional grafts have been noted to result in less regenerative potential with increasing graft lengths [2,9]; therefore, end-end repairs are the preferred surgical technique [10]. Unfortunately, end-end repairs are limited by the size and length of damaged nerve tissue. This study focuses on quantifying additional nerve length obtained after CPN neurolysis, with varying degrees of lower extremity flexion, to assist with end to end nerve repairs in the setting of nerve injury with significant gaps.

## Materials and methods

Dissections

For this study, full and partial internal neurolysis dissections were performed on salt- and formaldehyde-perfused cadavers. The fibular head was palpated for all dissections, and incisions were made to best mimic surgical conditions unless fixation conditions did not permit. If fixation did not permit joint mobility needed for the study, a small incision was made on the anterior thigh and the patellar tendon was transected. This incision was made to overcome stiffness from fixation and does not mirror clinical procedure. Although injury to peripheral nerves can occur anywhere along its course, a frequent CPN injury site is superficial at the knee joint, just proximal to the fibular head [5,8]. Therefore, all nerves were completely transected at the distal fascial border, just proximal to the fibular head, as the CPN passed deep to peroneus longus.

Full Internal Neurolysis

Full neurolysis was performed on seven legs of six formaldehyde-perfused cadavers and one salt cadaver. A linear incision was made on the posterior thigh from the fibular head to the inferior gluteal fold, disrupting the fascia lata enclosing the hamstring muscles. Using blunt dissection, the sciatic nerve was freed from the surrounding tissues (fascia, adipose, muscle, etc.) without transecting any branches to innervated muscles. Neurolysis started with a sub-gluteal dissection up to the sciatic notch of the pelvis. It continued inferiorly to the fibular head where the common peroneal nerve passed deep to peroneus longus. At the sciatic nerve's bifurcation, the branches were freed from surrounding tissues, and normal bifurcation anatomy for each cadaver was maintained. For the salt perfused cadaver, the patellar tendon remained intact due to flexibility.

Partial Internal Neurolysis

Partial neurolysis was performed on four legs of three salt-perfused cadavers, where a small incision was made from the bicep femoris tendon distally to the fibular neck. Unlike full internal neurolysis, no incision was made on the posterior thigh to liberate the sciatic nerve from surrounding tissues. The CPN was found as it passed inferiorly and superficially from the posterior aspect of biceps femoris tendon before diving deep to peroneus longus. Using blunt dissection, the tissue covering the superficial surface of the CPN was removed, but without disturbance to the leg's fascia composing compartments. Additional perpendicular incisions were made if cadaveric tissue did not permit proper execution in taking measurements. These incisions would not be performed under normal surgical conditions.

Measurements

Measurements of extra nerve were performed identically between full and partial internal neurolysis groups. The amount of nerve length gained from neurolysis without flexion was measured at the location of the CPN passing deep to peroneus longus before and after transection. Before being transected, the distal fascial border was identified and marked with a pin for consistency. Nerve length gained for both types of neurolysis was measured in cm at 10° increments using a protractor, starting at 90° and ending at 0°.

## Results

**Figure 1:**
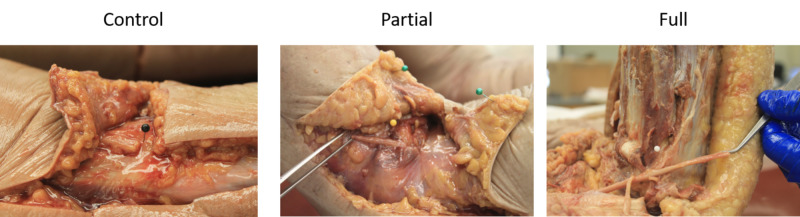
Salt body cadaver measurements of the common peroneal nerve for control, partial, and full neurolysis techniques. Example images display nerve length obtained after complete transection with full or partial internal neurolysis. Black, yellow, and white pins mark the distal fascial border where the common peroneal nerve (CPN) was transected before coursing deep to peroneus longus. Control neurolysis (left) shows transected nerve (partial neurolysis) with 0º of flexion. Partial neurolysis (middle) rests at 60º, full neurolysis (right) rests at 90º. Extra cuts present for mobility in order to take proper measurements and do not mimic surgical conditions. n= five-six legs/ dissection group

For the full neurolysis dissection group, the length of nerve obtained after neurolysis was consistent for formaldehyde- and salt-perfused cadavers, concluding that fixation did not affect measurements at any angle. Both formaldehyde- and salt-perfused cadavers have a positive correlation between the angle of measurement and length of nerve obtained after neurolysis (Figure 1). 

This statistically significant difference in nerve length is seen in both dissection groups with p-values <0.05 (Table 1).

**Table 1:** Mean additional nerve length obtained after full and partial neurolysis at each angle of measurement. The means were compared at each angle of measurement with a T-test and the p-value recorded. *= p<0.05, **= p<0.01, ***= p<0.001, two tailed T-test. n=5-6 legs/dissection group

Degrees	Dissection	Mean	P-Value
0	Full	0.42	0.04219 *
	Partial	0.00	
10	Full	0.79	0.00297 **
	Partial	0.30	
20	Full	1.25	0.002448 **
	Partial	0.55	
30	Full	1.71	0.006251 **
	Partial	0.95	
40	Full	2.58	0.001961 *
	Partial	1.40	
50	Full	3.33	0.001393 *
	Partial	1.95	
60	Full	4.33	0.0006015 ***
	Partial	2.25	
70	Full	5.17	0.0008892 ***
	Partial	2.40	
80	Full	5.75	0.0006533 ***
	Partial	2.80	
90	Full	6.83	0.000199 ***
	Partial	3.10	

**Figure 2:**
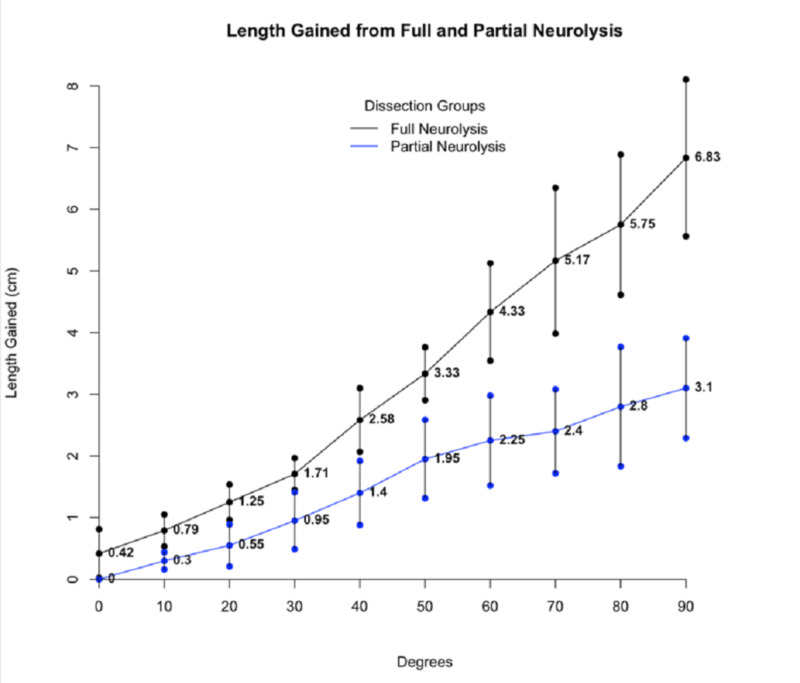
Mean additional nerve length Obtained after full neurolysis (black line and points) and partial neurolysis (blue line and points) with confidence intervals. n=5-6 legs/ dissection group.

Values were plotted with their confidence intervals (Figure 2), which did not overlap at any measurement angle between the two groups.

**Figure 3:**
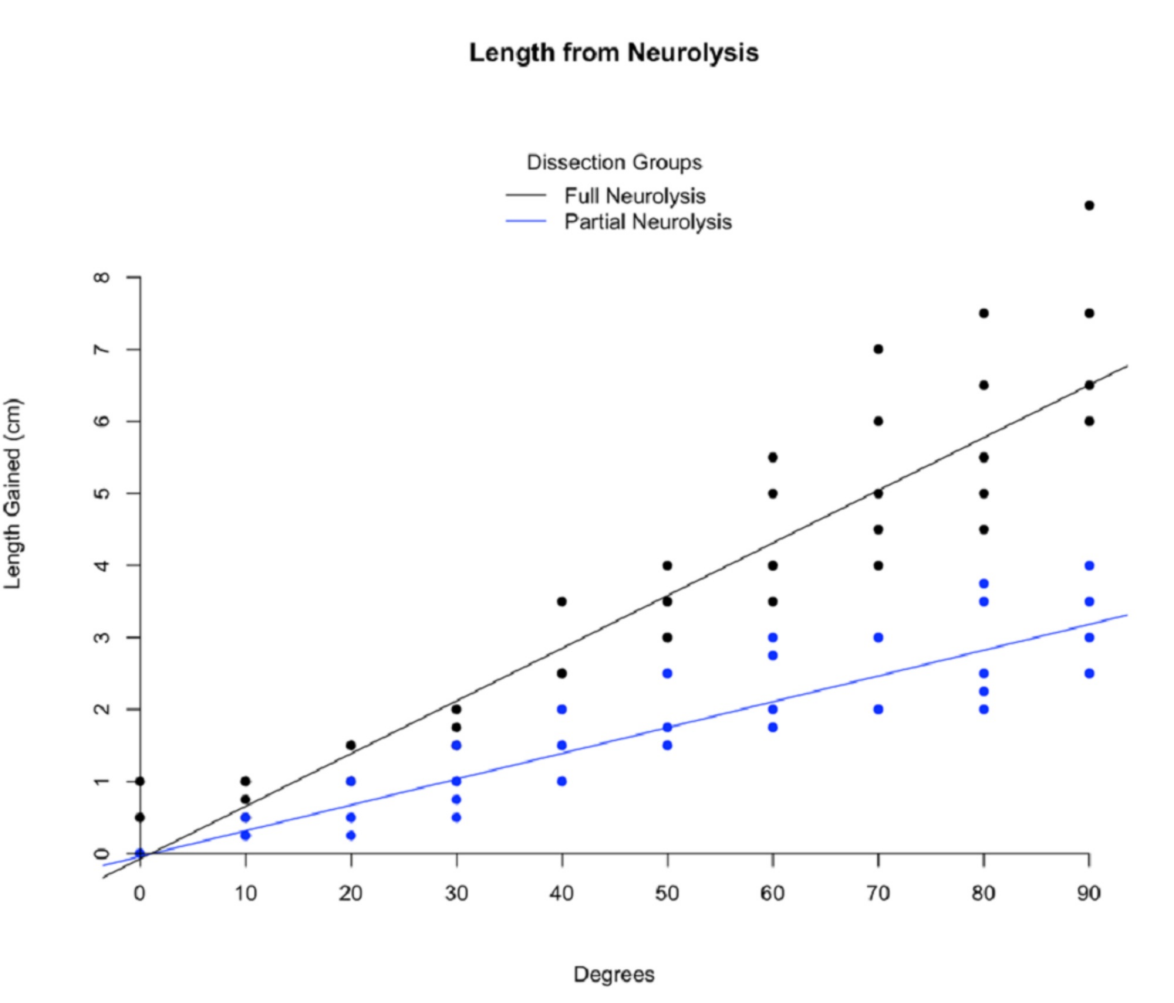
Scatterplot showing individual dissection measurements Each measurement performed is plotted with a line of best fit for each dissection group. n=5-6 legs/ dissection group.

The individual values were graphed on a scatterplot, and a linear regression was performed (Figure 3).

The full neurolysis group's R-squared value was 0.896, with an intercept of -0.074, a slope of 0.073, and a p-value <0.001. The R-squared value of the partial neurolysis group was 0.84, with an intercept of -0.042, a slope of 0.036, and a p-value <0.001. Descriptive statistics for the data sets, including mean values and 95% confidence intervals, were calculated and reported to two decimal places. A student's T-test was used to compare the mean values of the full and partial neurolysis groups at each measurement angle. Fixation was not a variable affecting neurolysis results; therefore, formaldehyde- and salt-perfused cadavers were grouped under dissection type. The level of statistical significance was set at p <0.05. All values were plotted, and a linear regression performed showed a correlation between the angle of measurement and the additional length of nerve obtained. Statistical analysis was performed using RStudio Version 1.1.463.

## Discussion

Injury to the common peroneal nerve can be caused by various mechanisms and currently accounts for 15% of all mononeuropathies in adults [11-13]. Due to its superficial course on the knee's posterolateral aspect, the CPN is highly susceptible to injury at the knee joint [8,9,11]. It has been reported to be damaged in up to 40% of cases following a multi-ligament knee injury, as well as during knee dislocations, leg fractures, and knee replacement surgeries [9,14,15]. Although some CPN injuries can resolve spontaneously without intervention, debilitating injuries resulting in motor dysfunction, sensory loss, scarring or compression, may require surgical intervention to prevent permanent damage. Previously, methods to repair significant nerve damage have resulted in nerve grafts, with some cases being studied to monitor post-operative care. The studies resulting from these cases have further concluded that nerve grafts exceeding 7 cm lead to significant decreases in motor function and patient recovery [4,9]. Recently, a study conducted in 2019 involving 20 patients, resulted in the average graft length for the CPN to be approximately 9$\pm$4 cm. In this study, only one patient with the shortest graft length (5 cm) regained motor function, while seven out of nine neurolysis patients reported satisfactory recovery [4]. Therefore, it is recorded that many CPN injuries result in injured segments greater than 7 cm. With end-end repairs being limited by gap size, this makes nerve grafting the only surgical technique available to rescue distal motor and sensory loss.

Secondly, our partial neurolysis group was designed to provide a less invasive approach to our neurolysis technique, in an attempt to mimic surgical conditions via a smaller incision that would minimize the risk of infection post-surgery. Although our full neurolysis group does not mimic the appropriate surgical conditions that other studies have used on patients, these clinical studies have performed internal and external neurolysis using techniques similar to our partial neurolysis group [8,16]. In these clinical studies, there was observed motor and sensory recovery after neurolysis, with one study concluding that out of 200 patients, only nine (4.5%) reported mild to severe complications after surgery [16]. Our study further strengthens these clinical studies' outcomes by including lower extremity flexion to increase the likelihood of direct end-end repairs. This provides an alternative technique for patients with significant nerve damage due to our full neurolysis group averaging 6.8 cm of additional nerve length at 90° of knee flexion (Table 1).

In conclusion, this technique can further increase nerve regeneration success along with motor and sensory functions across the injured segment, while also providing a positive impact on the patient's recovery and quality of life.

Study limitations and future directions

Limitations of this study include the partial neurolysis only being performed on salt perfused cadavers. Although not seen as a variable for the full neurolysis groups, to ensure the nerve length obtained from partial neurolysis is not dependent on the fixation method, formaldehyde cadavers are needed for partial neurolysis dissection group. Future directions of this study would be to perform partial neurolysis dissections on formaldehyde-perfused cadavers for confirmation and to use these data to incorporate neurolysis with knee manipulation more frequently in surgical end-end nerve repairs. 

## Conclusions

In this study, we found a direct statistically significant relationship between the extent of internal neurolysis (full vs partial) and the additional length of nerve obtained, despite fixation type. Although the fixation method didn’t alter results, salt preservation is the optimal cadaver specimen fixation, due to increased flexibility and mobility of the knee joint. The flexibility of cadaveric tissue under salt perfusion decreased the need for patella tendon cuts and extra incisions, therefore mirroring surgical conditions more precisely. We also found a direct relationship between the angle of measurement and the length of nerve obtained in both neurolysis groups. In summary, the greater the extent of neurolysis and knee flexion, the greater length of nerve obtained. These findings are clinically significant by allowing surgeons to perform more direct repairs, therefore minimizing graft lengths and increasing the success of nerve regeneration.
